# Preprints: Facilitating early discovery, access, and feedback

**DOI:** 10.1007/s40037-018-0451-8

**Published:** 2018-09-14

**Authors:** Lauren A. Maggio, Anthony R. Artino Jr, Erik W. Driessen

**Affiliations:** 1 Department of Medicine, F. Edward Hébert School of Medicine, Uniformed Services University of the Health Sciences, Bethesda, MD USA; 2 Faculty of Health, Medicine and Life Science, Maastricht University, Maastricht, The Netherlands

*Perspectives on Medical Education* preprint policy: PME encourages authors to deposit a preprint of their manuscript prior to submission for peer review on non-commercial servers such as bioRxiv, Open Science Framework Preprints, and SocARxiv. When submitting, authors must disclose in their cover letter that a version of the manuscript has been deposited as a preprint and include any associated DOIs or accession numbers. Upon publication in PME, authors are requested to update any preprint versions with a link to the final published article.

## What is a preprint?

A preprint is a complete version of a scientific manuscript that is uploaded to a preprint server by its authors prior to peer-review [1]. Preprints have been further described as ‘a ‘research output’ (report of original research, review, commentary, etc.) that has not completed a typical publication pipeline but is of value to the community and deserving of being easily discovered and accessed’ [2]. The majority of research outputs submitted as preprints are research articles, approximately 60% of which will ultimately be published in a peer-reviewed journal [3].

Preprint proponents have highlighted their value in relation to *early discovery, access,* and *opportunities for feedback*.

### Early discovery

In the biomedical sciences it takes on average 100 days from submission of a manuscript to acceptance [4]. In comparison, a preprint can be made available within a few hours to a few days. For many, including grant funders and researchers, this accelerated pace is appealing. For example in 2017, the National Institutes of Health noted that they ‘encourage investigators to use interim research products, such as preprints, to speed the dissemination and enhance the rigor of their work’ [5]. Other major funders, such as the European Research Council, Wellcome Trust, and the Canadian Institute of Health Research, have followed suit [6]. For researchers, the accelerated dissemination that preprints afford translates into opportunities to almost instantaneously share their research with potential collaborators and to demonstrate tangible evidence of achievement and productivity to potential funders and academic evaluators.

### Access

Unlike most of the scholarly literature [7], preprints are publicly accessible to the worldwide scientific community, which greatly enhances their overall visibility, thus ‘democratizing the flow of information’ [1]. To access a preprint, readers do not need to have a library password, pay a fee, or surrender their personal details. Instead, they can simply search the free, open-access preprint server for preprints. Notably, in some cases, a preprint may be the only publicly accessible version of a manuscript. For example, an author may deposit a publicly accessible preprint of their article, but ultimately publish the article in a subscription journal (access to which is often restricted behind a paywall). Preprints also provide authors an additional layer of access to meta-data about their work, such as the number of times an abstract has been viewed or the full-text downloaded. While some journals, such as *Perspectives on Medical Education,* make this information available, not all journals openly share these dissemination data.

### Opportunities for feedback

Most preprint servers are designed to enable the scientific community to comment and provide feedback on preprints. This capability provides authors with the chance to receive feedback early, when there is still opportunity for revision. In fields such as physics, providing feedback on preprints is a well-established procedure, which has been the field’s standard practice for over 20 years [1]. Additionally, feedback on preprints is not restricted to the select two or three peer reviewers and editors assigned to the manuscript inside a journal’s editorial manager. Instead, readers from multiple backgrounds, working from different philosophical stances and with different methods, can contribute new viewpoints and approaches. Some researchers have suggested that by facilitating such diverse feedback, preprints are an important component of open science practices that ultimately enable and improve the reproducibility and transparency of research. That is, ‘By making results more open earlier, they allow more eyes to scrutinize the results and find out errors or ways to improve the work before it gets disseminated as a published paper with a seal of formal publication’ [8].

Despite the above benefits, critics have raised concerns about preprints. For example, there is concern that readers, including scientists, journalists, and the lay public, will not recognize that a preprint has not been peer reviewed and possibly act on unexamined and potentially invalid evidence. For scientists this could lead to citing unvetted research thus compromising the integrity of the literature base. However, Lariviere and colleagues found that only between 0.2 to 6.6% of preprints deposited in arXiv, depending on specialty, were cited and that those numbers are declining [9]. Another concern is that preprint servers may become ‘dumping grounds’ for poor-quality research [8]. However, an analysis of almost 8000 preprints deposited to bioRxiv found that over half were published in peer-reviewed journals within 12 months of deposit [3].

## Preprints in health professions education

While increasingly common in the bio and clinical sciences—over 5000 manuscripts were deposited in the life science repository bioRxiv (pronounced ‘bio-archive’) last year alone [10]—depositing preprints is a new practice in health professions education (HPE). Therefore, to contextualize and encourage this practice in HPE, we share our personal preprint experience.

In January 2018, we completed a manuscript reporting survey results on questionable research practices in HPE. In our analysis, we discovered areas of concern for the HPE research community and thus wanted to communicate those concerns to the community quickly. As a research team, we explored the possibility of depositing our manuscript as a preprint.

To begin, we emailed the editor-in-chief of the journal to which we planned to submit. In the email, we described our rationale and provided links to information about preprints and examples from bioRxiv. The editor discussed our request with his editorial team and shortly thereafter granted us permission to deposit our preprint. We then submitted the article to the journal and, in our submission cover letter, also noted our intention to deposit our article as a preprint.

Following the editor’s approval and our submission to the journal, we submitted our preprint to bioRxiv in the ‘scientific communication and education’ category. (Note: generally authors deposit a preprint *prior* to submitting to their journal of choice. Our experience varies slightly due to the newness of preprints in HPE and our interactions with the journal’s editorial team). Following a basic screening process to check for plagiarism and dangerous or offensive content, our preprint was posted and assigned a unique digital object identifier (DOI) after approximately 48 h [11]. Simultaneously, bioRxiv tweeted a link to our preprint to its over 18,000 followers. Over the next five months, the abstract of the preprint was viewed over 2300 times and the full-text downloaded more than 1200 times. Additionally, the preprint has been tweeted 58 times and discussed on three blogs. On social media, the HPE community started a conversation about our article’s content and also the preprint process itself. Additionally, beyond HPE, researchers in mathematics, ecology, biochemistry, law, and sociology have discussed and shared our work. In June 2018, our manuscript was officially accepted for publication by the journal.

As an author team, we feel the benefits of posting our manuscript as a preprint were many. Most immediately, it enabled us to open an important conversation on social media about questionable research practices in HPE, a conversation that crossed over and was discussed by researchers in several other fields. From a motivational standpoint as researchers, disseminating our work was fun! We found it exhilarating to disseminate our findings while they were still ‘fresh’ and while we were still excited about the research (as opposed to waiting months to get the word out). Next, we were able to reference our preprint on a grant proposal to continue expanding our related program of research on the responsible conduct of research. Moreover, we got a ‘time stamp’ on our new findings, since bioRxiv provides a posting date and DOI, thus giving us the opportunity to ‘plant our flag in the ground’ on this important research topic. Finally, our preprint stimulated collaboration. Since posting to bioRxiv, we have heard from several HPE scientists interested in collaborating, and we have started a follow-on project exploring authorship practices.

Recently, our article on questionable research practices in HPE was accepted by the peer-reviewed journal mentioned above, after a round of major revisions. Once the journal makes the published ahead of print version available online, a link will be included on our preprint’s web page, alerting readers and directing them to the updated, peer-reviewed version of the article.

From our perspective, posting a preprint and submitting to a traditional journal gave us the best of both worlds: we gained valuable feedback from the reviewers and editors, and made important revisions through the peer-review process, and yet by posting a preprint we were able to take advantage of early discovery, access, and opportunities for feedback (from outside the community).

## Preprints in *Perspectives on Medical Education*

To facilitate early discovery, access, and feedback to research and scholarship, *Perspectives on Medical Education* invites authors to deposit a preprint of their manuscript in a non-commercial preprint server prior to submission to the journal. (See below for PME’s preprint policy). As an initial foray into preprints, the journal has published its first article that was recently deposited as a preprint prior to submission to the journal [12]. In the future, we look forward to supporting open science practices and receiving more article submissions that have been deposited as preprints.

### Disclosures

Written work prepared by employees of the Federal Government as part of their official duties is, under the US Copyright Act, a ‘work of the United States Government’ for which copyright protection under Title 17 of the United States Code is not available. As such, copyright does not extend to the contributions of employees of the Federal Government.

The views expressed in this article are those of the authors and do not necessarily reflect the official policy or position of the Uniformed Services University of the Health Sciences, the U.S. Navy, the Department of Defense, or the U.S. Government.
